# Supplementary material for: Complexome profiling of the Chlamydomonas psb28 mutant reveals TEF5 as an early PSII assembly factor
Source: Plant Cell. 2025 Mar 17;37(6):koaf055. doi: 10.1093/plcell/koaf055 (PMC12164590; doi:10.1093/plcell/koaf055)
Supplement: koaf055_Supplementary_Data [file koaf055_supplementary_data.zip › psb28_interactive.zip!/plots/intensity/qualityPlots/peptides/CGLD38_Cre13.g562750.t1.2.html]

### Cre13.g562750.t1.2

Trivial name: CGLD38  
SeqCoverage [median]: 17.00  
Mass: 64.02 kDa  
  
 

| Sequence | MissedCleavage | Mass [Da] | wtIntensity | muIntensity | Score |
| --- | --- | --- | --- | --- | --- |
| AAEAEAAAEAAAAEADGEAAK | 0 | 1886.85 | 0.00 | 1985.82 | 87.49 |
| AEVAAIARPEPVEDSR | 1 | 1708.87 | 1722.57 | 1232.08 | 82.10 |
| KPPAQPAQPAK | 1 | 1131.64 | 1320.36 | 477.91 | 62.63 |
| LVAAPAPAGPGGVGPRPVVFPETDMK | 1 | 2529.34 | 0.00 | 1104.91 | 54.22 |
|  |  |  | 3042.9 | 4800.7 |  |
